# Everolimus as maintenance therapy in advanced neuroendocrine neoplasms: results from the MAVERIC phase II trial

**DOI:** 10.1093/oncolo/oyaf432

**Published:** 2026-01-22

**Authors:** Lorenzo Antonuzzo, Daniele Lavacchi, Francesca Spada, Riccardo Marconcini, Fabio Gelsomino, Vito Amoroso, Federica Cosso, Elisa Pellegrini, Federico Scolari, Clotilde Sparano, Giulia Massaro, Elisa Giommoni, Luca Messerini, Daniele Rossini, Marco Brugia, Francesco Di Costanzo, Luca Boni, Massimo Milione, Serena Pillozzi, Nicola Fazio, Gruppo Oncologico Italiano di Ricerca Clinica (GOIRC)

**Affiliations:** Oncology Unit, Careggi University Hospital, Florence 50134, Italy; Department of Experimental Clinical Medicine, University of Florence, Florence 50134, Italy; Oncology Unit, Careggi University Hospital, Florence 50134, Italy; Division of Gastrointestinal Medical Oncology and Neuroendocrine Tumors, European Institute of Oncology (IEO), IRCCS, Milan 20141, Italy; Medical Oncology, Dipartimento di Oncologia Medica, AOU Pisana—Stabilimento di Santa Chiara, Pisa 56126, Italy; Department of Oncology and Hematology, Division of Oncology, University Hospital of Modena, Modena 41125, Italy; Medical Oncology Unit, Azienda Socio-Sanitaria Territoriale (ASST) Spedali Civili di Brescia, Genova 16132, Italy; Department of Experimental Clinical Medicine, University of Florence, Florence 50134, Italy; Oncology Unit, Careggi University Hospital, Florence 50134, Italy; Department of Experimental and Clinical Biomedical Sciences, University of Florence, Genova 16132, Italy; Department of Experimental and Clinical Biomedical Sciences, University of Florence, Genova 16132, Italy; Department of Experimental Clinical Medicine, University of Florence, Florence 50134, Italy; Oncology Unit, Careggi University Hospital, Florence 50134, Italy; Department of Experimental Clinical Medicine, University of Florence, Florence 50134, Italy; Oncology Unit, Careggi University Hospital, Florence 50134, Italy; Department of Experimental Clinical Medicine, University of Florence, Florence 50134, Italy; Oncology Unit, Careggi University Hospital, Florence 50134, Italy; Oncology Unit, Careggi University Hospital, Florence 50134, Italy; Clinical Epidemiology Unit, IRCCS Ospedale Policlinico San Martino, Genova 16132, Italy; First Pathology Unit, Department of Pathology and Laboratory Medicine, Fondazione IRCCS Istituto Nazionale dei Tumori, Milan 20133, Italy; Department of Experimental and Clinical Biomedical Sciences, University of Florence, Genova 16132, Italy; Division of Gastrointestinal Medical Oncology and Neuroendocrine Tumors, European Institute of Oncology (IEO), IRCCS, Milan 20141, Italy

**Keywords:** metastatic neuroendocrine neoplasms, everolimus, maintenance therapy

## Abstract

**Background:**

Neuroendocrine neoplasms (NEN) are a heterogeneous disease and chemotherapy (CT) represents the standard first-line treatment for those with a Ki-67 index >20%.

**Methods:**

MAVERIC is a randomized, multicenter, non-comparative phase II study including patients with metastatic gastroenteropancreatic (GEP-NEN) or large-cell neuroendocrine carcinoma (LCNEC) (Ki-67 20%-55%) according to the 2010 WHO grading system and at least stable disease after first-line CT. Patients were randomized (2:1) to everolimus 10 mg/day or observation until progression or treatment intolerance. The primary endpoint was progression-free survival (PFS); secondary endpoints included overall survival (OS) and safety.

**Results:**

Between November 2015 and June 2022, 30 patients were enrolled across 5 Italian centers, with 20 assigned to everolimus and 10 to observation. The analysis included 29 patients (52% GEP-NEN, 48% LCNEC). Median (m)PFS was 11.8 months in the everolimus arm and 1.8 months in the control arm. MOS was similar between arms (38.3 and 38.2 months). The subgroup of 11 patients with GEP-neuroendocrine tumor (NET) grade 3 treated with everolimus showed an mPFS of 19.9 months and mOS of 48.1 months. Most common adverse events (AEs) were mucositis (80%), dyslipidemia (55%), fatigue (45%), pneumonitis (40%), and peripheral edema (35%). Grade 3 AEs occurred in 70% of patients; no grade 4 AEs were observed.

**Conclusions:**

The MAVERIC trial demonstrated encouraging clinical benefit of everolimus in metastatic GEP-NEN and LCNEC (Ki-67 20%-55%) following first-line CT. Toxicity was consistent with the known safety profile of everolimus. This strategy was particularly effective in GEP-NEN patients and warrants further investigation. ClinicalTrials.gov identifier: NCT02687958 (https://clinicaltrials.gov/study/NCT02687958).

Lessons Learned

- Everolimus demonstrated encouraging clinical benefit in patients with metastatic GEP-NEN or LCNEC with Ki-67 index between 20% and 55% as maintenance treatment following standard chemotherapy with a manageable safety profile
- In NENs with a Ki-67 index >20% the optimal CT regimen and duration remain undefined, and no maintenance therapy is currently indicated.

## Background

Emerging evidence has progressively challenged the concept of high-grade neuroendocrine neoplasm (NENs), and in responsive patients after first-line chemotherapy (CT) a standard approach has not been defined yet.^1^^,^^2^

Everolimus, an oral inhibitor of mammalian target of rapamycin (mTOR), could have a potential role in this setting although limited data are available.^3^

## Trial information

**Figure 1.**
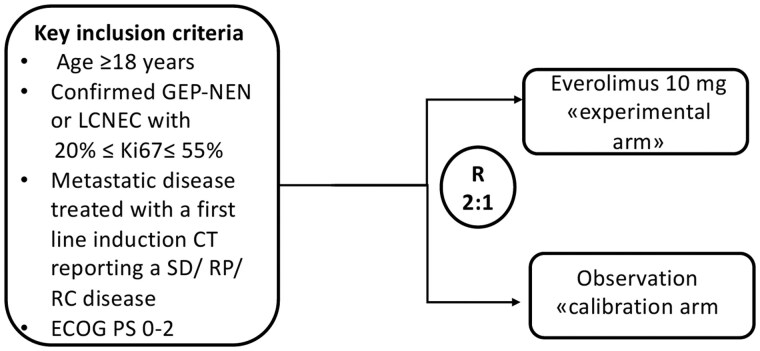
Flow chart of MAVERIC trial. Patients with metastatic GEP-NEN or LCNEC (Ki-67 20%-55%) according to the 2010 WHO classification achieving at least SD after first-line CT that were randomized (2:1) to everolimus 10 mg/day or observation. GEP-NEN, gastroenteropancreatic neuroendocrine neoplasm; LCNEC, lung large cell neuroendocrine carcinoma; SD, stable disease; RP, partial response; RC, complete response; R, randomization.

MAVERIC was a multicenter, randomized, open-label, non-comparative, phase II clinical trial performed at 5 Italian institutions. The main inclusion criteria were an age of 18 years or older; histologically or cytologically confirmed, advanced, non-functioning, gastroenteropancreatic (GEP)-NEN) or large-cell neuroendocrine carcinoma (LCNEC) of lung origin with Ki-67 index ranging from 20% and 55% in accordance with the 2010 World Health Organization (WHO) classification^4^ (Figure 1); measurable target lesions according to the Response Evaluation Criteria in Solid Tumors (RECIST) guideline^5^; evidence of stable disease (SD), partial response (PR) or complete response after first-line CT according to clinical practice; Eastern Cooperative Oncology Group (ECOG) performance status ≤ 2; adequate organ function. The main exclusion criteria were well differentiated (WD) NEN with Ki67 < 20% or neuroendocrine carcinoma with Ki-67 > 55%; functional disease; concurrent mutually exclusive drugs with everolimus; presence of brain metastases. Histology performed before 2017 has been revised and classified according to the latest WHO classification by each reference center.

The study was done in accordance with Good Clinical Practice guidelines, the ethical principles of the Declaration of Helsinki, and local regulatory requirements. (EudraCT registration number is 2014-003951-72). All patients provided written informed consent before enrolment.

The primary endpoint was radiology-assessed progression-free survival (PFS), defined as the time between randomization and the first evidence of progressive disease (PD) or death, whichever occurred first. Documentation of PD was defined as per RECIST version 1.1 criteria^5^ based on investigator-reported evaluation. Overall survival (OS), defined as the time from randomization to the date of death due to any cause, and safety were the main secondary endpoints (Table 1).

**Table 1.** Trial information.

Trial information		
**Disease**		Neuroendocrine neoplasm
**Stage of disease/treatment**		Metastatic/advanced
**Prior therapy**		1 prior regimen achieving at least stable disease according to RECIST v1.1 criteria.
**Type of study**		Phase II
**Primary endpoints**		Progression-free survival
**Secondary endpoints**		Overall survival and safety
**Exploratory analysis**	Tumoral tissue and blood samples were collected for further investigations exploring prognostic and predictive factors.	

Adverse events (AE) were assessed as per National Cancer Institute Common Terminology Criteria for AE (CTCAE) version 4.03.

## Drug information

Patients were randomly assigned with a 2:1 ratio to maintenance therapy with everolimus 10 mg per day (experimental arm) or to observation (control arm) until progression or toxicity (Table 2).

**Table 2.** Drug information.

Drug information	
**Arm**	Experimental
**Generic/working name**	Everolimus
**Company name**	Novartis Pharmaceuticals Corporation
**Drug type**	Protein kinase inhibitor
**Drug class**	mTOR (mammalian target of rapamycin) kinase inhibitors
**Dose**	10 mg
**Route**	Oral
**Schedule of administration**	Once daily

Randomization was done using a centralized web-based system and allocation sequence was masked and generated at the Clinical Trials Coordinating Center, Oncology Unit, Careggi University Hospital (Florence, Italy).

The time between the last cycle of CT and randomization must not exceed 28 days.

All patients who underwent randomization were assessed for efficacy by radiological imaging every 12 weeks. Visits and study drug dispensation occurred in cycles, with each cycle equaling 28 days. Dose reductions and treatment interruption were allowed for patients who did not tolerate therapy or to manage adverse events that were judged to be related to study treatment. Two dose reductions were allowed: from 10 mg to 5 mg per day and, subsequently, to 5 mg every other day.

Palliative radiotherapy was allowed.

## Statistical analysis

This is an open-label phase II study not powered for statistical comparison between experimental and observation arm. A control arm was included to provide contextual and calibration data due to lack of historical data for comparison in this subgroup of patients.

Survival analyses were descriptive. Kaplan–Meier methods were used to estimate median (m)PFS and OS with corresponding 95% CIs. No formal hypothesis testing between groups was performed. All statistical analyses were conducted using the R software v4.3.0 and the packages survival v3.5-5, survminer v0.4.9, and dplyr v1.1.2, and SAS (version 9.4).

## Patient characteristics

**Figure 2.**
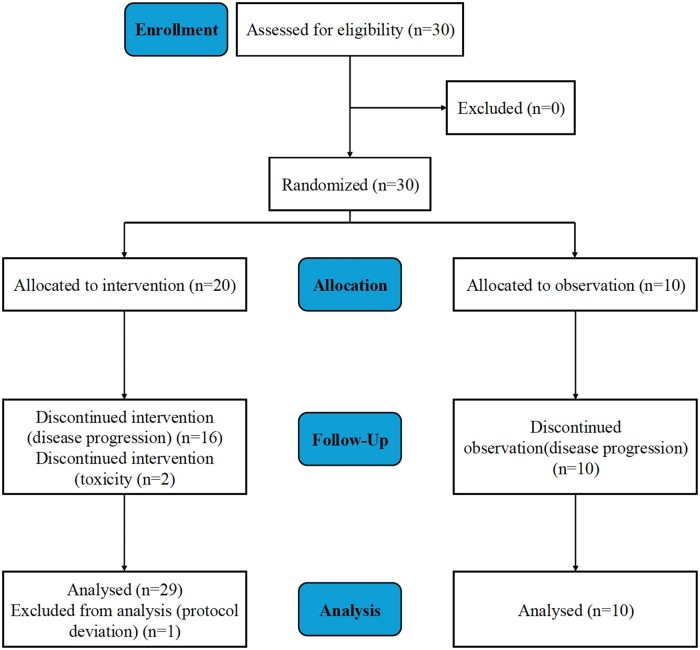
Consort flow diagram.

From November 2015 to June 2022, 30 patients with metastatic, non-functioning, NEN from GEP or lung primary sites and Ki-67 index 20%-55% were enrolled and randomly assigned to everolimus (20 patients) or observation (10 patients) (Figure 2). One patient was not included in the analysis for protocol deviation having a Ki-67 > 55%. Patients’ baseline characteristics are reported in Table 3. The primary site was GEP in 52%, mostly pancreatic, while 48% of lung origin that resulted equally distributed in the 2 arms. WD neuroendocrine tumors (NETs) were more common in the experimental arm (85% of patients) than in the control arm (55%). Prior first-line CT was platinum-based in 13 patients and non-platinum-based in 16 patients, with 4 median number of CT cycles in both arms. A PR was achieved in 67% of patients in the control arm, with an equal distribution among platinum and non-platinum-based CT and in 35% of patients in the experimental arm, 60% with non-platinum-based CT.

**Table 3.** Patient characteristics.

**Patient characteristics**			
	Entire cohort (*n* = 29)	Treatment arm (*n* = 20)	Control arm (*n* = 9)
**Sex**			
**Male**	10 (35%)	5 (25%)	5 (55%)
**Female**	19 (65%)	15 (75%)	4 (45%)
**Median age (years)**	63 (38-84)	64 (38-81)	61 (52-84)
**ECOG performance status**			
**0-1**	28 (97%)	20 (100%)	8 (89%)
**2**	1 (3%)	0 (0%)	1 (11%)
**Prior first-line surgery**			
**Yes**	8 (28%)	6 (30%)	2 (22%)
**No**	21 (72%)	14 (70%)	7 (78%)
**Primary site**			
**Lung**	14 (48%)	7 (35%)	7 (78%)
**GEP**	15 (52%)	13 (65%)	2 (22%)
**Histologic grade**			
***Well differentiated***	22 (76%)	17 (85%)	5 (56%)
**GEP origin *(8 pancreatic, 3 small bowel, and 2 rectum origin)***	13 (45%)	11 (55%)	2 (22%)
**Lung origin**	9 (31%)	6 (30%)	3 (33%)
***Poorly differentiated***	7 (24%)	3 (15%)	4 (44%)
**GEP origin *(pancreatic)***	2 (7%)	2 (10%)	0 (0%)
**Lung origin**	5 (17%)	1 (5%)	4 (44%)
**2010 WHO grading system^a^**			
**GEP NEC G3**	15 (52%)	13 (65%)	2 (22%)
**LCNEC**	14 (48%)	7 (35%)	7 (78%)
**2017 WHO grading system^b^**			
**GEP NET G3**	13 (45%)	11 (55%)	2 (22%)
**GEP NEC G3**	2 (7%)	2 (10%)	0 (0%)
**LCNEC**	14 (48%)	7 (35%)	7 (78%)
**%Ki67**			
**20-29**	12 (41%)	9 (45%)	3 (33%)
**30-55**	17 (59%)	11 (55%)	6 (67%)
**Metastasis sites**			
**1**	7 (24%)	7 (35%)	0 (0%)
**>1**	22 (76%)	13 (65%)	9 (100%)
**First-line chemotherapy**			
**Platinum-based (carboplatin + etoposide, cisplatin + etoposide, cisplatin, FOLFOX)**	13 (45%)	8 (40%)	5 (56%)
**Non-platinum-based (capecitabine + temozolomide, capecitabine, temozolomide)**	16 (55%)	12 (60%)	4 (44%)
**First-line chemotherapy response**			
**SD**	16 (55%)	13 (65%)	3 (33%)
**PR**	13 (45%)	7 (35%)	6 (67%)
**Functional staging positivity**			
**Yes—Octreoscan**	2 (10%)	1 (7%)	1 (17%)
**Yes—PET (68) Gallium**	13 (65%)	9 (64%)	4 (66%)
**No**	5 (25%)	4 (29%)	1 (17%)
**Not done**	9	6	3
**Metabolic staging positivity**			
**Yes**	14 (88%)	7 (78%)	7 (100%)
**No**	2 (12%)	2 (22%)	0 (0%)
**Not done**	13	11	2
**Concomitant somatostatin analog**			
**Yes**	8 (28%)	7 (35%)	1 (11%)
**No**	21 (72%)	13 (65%)	8 (89%)

Data are *n* (%) or median (range). Percentages are calculated among patients with non-missing data.

a Patients assigned according to the 2010 WHO grading system.

b Patients assigned according to the updated 2017 WHO grading system.

### Primary assessment method

**Figure 3.**
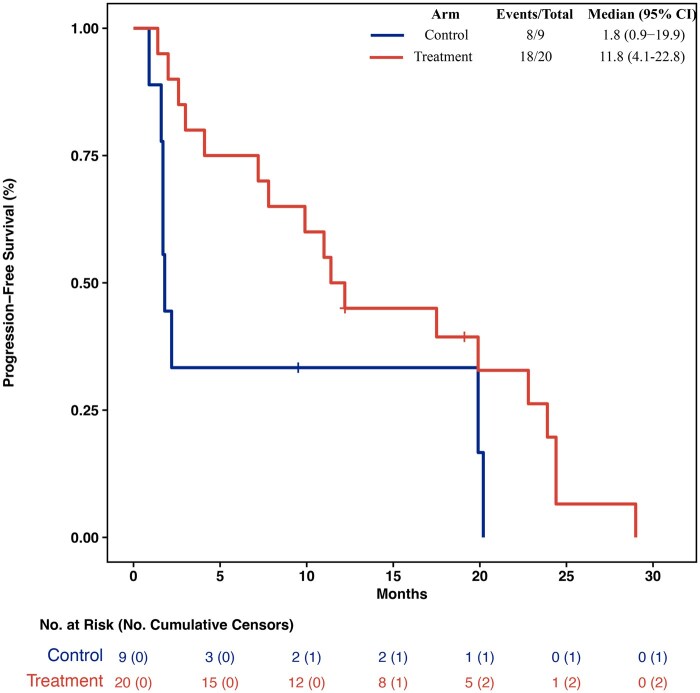
Kaplan–Meier curves and risk table of progression-free survival (PFS) in the control and treatment arm. NA, not reached.

After a median follow-up of 80 months, mPFS was 11.8 months (95% CI 4.1-22.8) in the experimental and 1.8 months (95% CI 0.9-19.9) in the control arm (Figure 3 and Table 4). MOS were 38.3 months (95% CI 14.9-59.6) and 38.2 months (95% CI 3.1-64.5), respectively.

**Table 4.** Primary assessment method.

Primary assessment method	
**Title**	Disease control (median progression-free survival).
**Number of patients screened**	30
**Number of patients enrolled in the experimental arm**	20
**Number of patients evaluable for toxicity**	19
**Number of patients evaluated for efficacy**	19
**Evaluation method**	Objective tumor assessments evaluated by imaging every 12 weeks of the initial randomized period according to RECIST v1.1 criteria.
**Outcome notes**	Kaplan–Meier graph is provided in Figures 3

**Figure 4.**
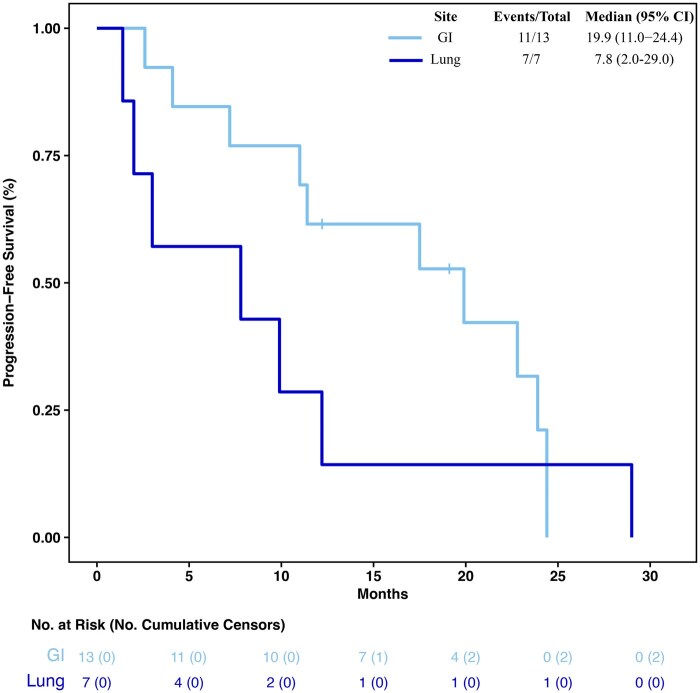
Kaplan**–**Meier curves and risk table of progression-free survival (PFS) in the GI and lung subgroups of the treatment arm. GI, gastrointestinal; NA, not reached.

In the 13 GEP-NEN patients treated with everolimus, the mPFS was 19.9 months (95% CI 11.0-24.4) and the mOS 48.1 months (95% CI 34.5-NA). Among the 7 patients with LCNEC, mPFS was 7.8 months (95% CI 2.0-29.0) (Figure 4) and mOS 14.6 months (95% CI 3.8-59.6).

**Figure 5.**
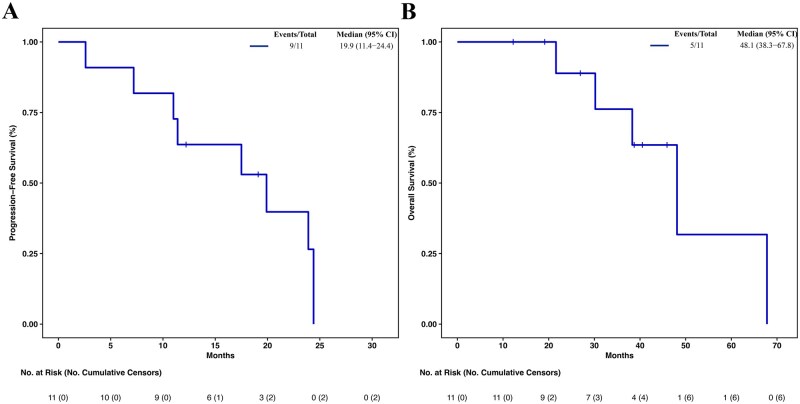
Kaplan**–**Meier curves and risk tables of progression-free survival (PFS) (A) and overall survival (OS) (B) in the GI NET G3 population of the treatment arm. GI, gastrointestinal; NET, neuroendocrine tumors; NA, not reached.

GEP-NENs specimens were reviewed by an expert pathologist in accordance with the 2017 WHO classification and 86% were reclassified as NET G3. The subgroup of 11 patients with GEP NET G3 treated with everolimus showed an mPFS of 19.9 months (95% CI 11.4-24.4) and mOS of 48.1 months (95% CI 38.3-67.8) (Figure 5).

### General toxicity profile

Drug-related AEs in the experimental arm were in line with the known safety profile of everolimus and were mostly grade (G) 1 or 2 (Table 5). The most common AEs reported in patients receiving everolimus were mucositis, dyslipidemia, fatigue, pneumonitis and peripheral edema. Globally, 14 patients (70%) experienced a G3 AE and 2 of them were classified as serious AE with consequent discontinuation of treatment (Table 6). In 65% of patients, at least one dose reduction was needed. No G4 AEs occurred.

**Table 5.** Drug-related adverse events in at least 10% of patients.

Drug-related adverse events in at least 10% of patients		
Adverse event	Grade 1 or 2 no. of patients (%)	Grade 3 no. of patients (%)
**Mucositis**	12 (60)	4 (20)
**Dyslipidemia**	9 (45)	2 (10)
**Asthenia/fatigue**	8 (40)	1 (5)
**Pneumonitis**	7 (35)	1 (5)
**Peripheral edema**	6 (30)	1 (5)
**Diarrhea**	6 (30)	0 (0)
**Neutropenia**	5 (25)	2 (10)
**Anemia**	5 (25)	1 (5)
**Thrombocytopenia**	5 (25)	0 (0)
**Nausea**	4 (20)	0 (0)
**Hyperglycemia**	3 (15)	1 (5)
**Xerostomia and xerodermia**	3 (15)	0 (0)
**Dysgeusia**	3 (15)	0 (0)
**Leucopenia**	3 (15)	0 (0)
**Cough**	3 (15)	0 (0)
**Hypertransaminasemia**	3 (15)	0 (0)
**Pyrexia**	3 (15)	0 (0)
**Rash**	2 (10)	1 (5)
**Pruritus**	2 (10)	0 (0)
**Hand-foot syndrome**	2 (10)	0 (0)

**Table 6.** Serious adverse events.

Serious adverse events		
**Dose level**	**Adverse event(s), grade**	**Attribution**
**Interruption**	Mucositis G3	Possible
**Interruption**	Sepsis G3	Not related

## Discussion

The MAVERIC trial focused on a particular subgroup of high-grade NENs, with Ki-67 between 20% and 55%, including both well and poorly differentiated NENs defined according to the WHO 2010 classification criteria.

Current guidelines indicate CT as first-line treatment in high-grade NENs although evidence remains limited to specific schedule or duration, particularly for advanced GEP NET G3.^6^ Similarly, clinical data on everolimus in this setting are also limited. The association of TEM and everolimus was assessed as upfront therapy in GEP-NET G3 and GEP-NEC reporting an mPFS of 12.6 months and 3.4 months, respectively, which are consistently in line with MAVERIC efficacy outcomes, with a comparable toxicity profile.^7^

Notably, unlike Morken’s study, MAVERIC trial evaluated everolimus as a maintenance treatment following first-line CT, which, to our knowledge, represents the first in this distinct patient population. Everolimus prolonged PFS suggesting that a biological therapy could replace CT to keep a longer tumor response.

The apparent lack of OS benefit with everolimus in our study (ie, 38.3 vs 38.2 months) may reflect the high percentage of patients receiving second-line therapy in the control arm at the time of progression (7/9 patients, 78%). Considering the high-grade lung NEN, the further rarity of this subtype implies even more uncertainties. In our trial, 7 patients in the everolimus arm had LCNEC with an mPFS of 7.8 months and mOS of 14.6 months, confirming the worse outcome of this population. Recently, a retrospective series of carcinoids with Ki-67 > 20% has reported a favorable response to both temozolomide (*n* = 12, PFS 6.8 months) and everolimus (*n* = 8, PFS 12.1 months) regimes, indirectly supporting our findings.^8^

Considering the OS in high-grade NEN, the present cohort shows a prolonged mOS which results almost doubled compared to literature data.^9^^,^^10^ This survival gap could be affected by several factors, such as the good baseline prognostic characteristics, the restricted inclusion criteria, and the use of different treatment lines after disease progression.

In our study, several limitations should be recognized. Considering the rarity of these neoplasms, the enrollment lasted almost 7 years, and different primary sites were included; the WHO classification has been updated during the accrual period and consequentially most of LC-NECs (9/14) were retrospectively reclassified as WD. In WD lung or GI NETs, although limited to progressive disease, the activity of everolimus has been also reported in RADIANT-4, which is consistent with our results.^3^ Moreover, during study’s accrual, first-line 177Lu-DOTATATE data for high G2 and G3 SSTR+ GEP NETs was not available.^11^ Despite the reported limitations this study discloses several strengths: the therapeutic approach has shed light on a specific sequential treatment; although small, the population sample represents an authentic overview of advanced NEN, joining 2 different aggressive cohorts of high-grade lung and GEP-NEN, to explore the therapeutic benefits of the same treatment scheme.

In conclusion, the MAVERIC trial is a hypothesis generating study, showing that everolimus may be effective and safe in patients with advanced non-functional GEP-NEN and LCNEC with a Ki-67 index ranging from 20% and 55% as maintenance therapy after a first-line CT. This strategy was particularly effective for the GEP-NEN patients.

## Data Availability

The datasets generated and/or analyzed during the present study are available upon reasonable request to the corresponding author.
